# Co-treatment with interferon-γ and 1-methyl tryptophan ameliorates cardiac fibrosis through cardiac myofibroblasts apoptosis

**DOI:** 10.1007/s11010-019-03542-7

**Published:** 2019-04-22

**Authors:** Jun-Won Lee, Ji Eun Oh, Ki-Jong Rhee, Byung-Su Yoo, Young Woo Eom, Sang Wook Park, Ji Hyun Lee, Jung-Woo Son, Young Jin Youn, Min-Soo Ahn, Sung-Gyun Ahn, Jang-Young Kim, Seung-Hwan Lee, Junghan Yoon

**Affiliations:** 1 Department of Internal Medicine, Yonsei University Wonju College of Medicine, 20 Ilsan-ro, Wonju, Gangwon-do 26426 Korea; 2 Cell Therapy and Tissue Engineering Center, Yonsei University Wonju College of Medicine, 20 Ilsan-ro, Wonju, Gangwon-do 26426 Korea; 3 Cell Department of Biomedical Laboratory Science, Yonsei University College of Health Sciences, Wonju, Korea

**Keywords:** Myofibroblast, Interferon-gamma, Indoleamine 2,3-dioxygenase, Apoptosis

## Abstract

Cardiac remodeling characterized by cardiac fibrosis is a pathologic process occurring after acute myocardial infarction. Fibrosis can be ameliorated by interferon-gamma (IFN-γ), which is a soluble cytokine showing various effects such as anti-fibrosis, apoptosis, anti-proliferation, immunomodulation, and anti-viral activities. However, the role of IFN-γ in cardiac myofibroblasts is not well established. Therefore, we investigated the anti-fibrotic effects of IFN-γ in human cardiac myofibroblasts (hCMs) in vitro and whether indoleamine 2,3-dioxygenase (IDO), induced by IFN-γ and resulting in cell cycle arrest, plays an important role in regulating the biological activity of hCMs. After IFN-γ treatment, cell signaling pathways and DNA contents were analyzed to assess the biological activity of IFN-γ in hCMs. In addition, an IDO inhibitor (1-methyl tryptophan; 1-MT) was used to assess whether IDO plays a key role in regulating hCMs. IFN-γ significantly inhibited hCM proliferation, and IFN-γ-induced IDO expression caused cell cycle arrest in G0/G1 through tryptophan depletion. Moreover, IFN-γ treatment gradually suppressed the expression of α-smooth muscle actin. When IDO activity was inhibited by 1-MT, marked apoptosis was observed in hCMs through the induction of interferon regulatory factor, Fas, and Fas ligand. Our results suggest that IFN-γ plays key roles in anti-proliferative and anti-fibrotic activities in hCMs and further induces apoptosis via IDO inhibition. In conclusion, co-treatment with IFN-γ and 1-MT can ameliorate fibrosis in cardiac myofibroblasts through apoptosis.

## Introduction

Cardiac remodeling is a pathologic process leading to structural and functional derangement of damaged cardiac tissue after acute myocardial infarction [1]. The severity of cardiac remodeling is mainly determined by the extent of tissue infarction and the degree of cardiac repair. Cardiac fibrosis is a representative response to various pathophysiological cardiac conditions. It is characterized by the excessive production and accumulation of extracellular matrix (ECM) components (consisting of collagen, fibronectin, glycosaminoglycan, and elastin) in the injured cardiac tissue [2]. This cellular change causes increased stiffness with decreased compliance of the heart, resulting in both systolic and diastolic dysfunction. Cardiac repair requires a balanced healing process from the early inflammatory phase to the reparative and proliferative phases [1, 3]. Appropriate induction and proliferation of cardiac myofibroblasts (CMs) help to prevent cardiac rupture with scar formation, though ongoing pathological activation of CMs causes fibrotic changes to the non-infarcted surrounding tissues followed by a deterioration of cardiac function [4–11]. One potential therapeutic approach is to inhibit the progression of cardiac fibrosis. CMs are widely recognized as a key factor mediating cardiac fibrosis because activated CMs can produce excessive ECMs and express the highly contractile protein α-smooth muscle actin that remodels the surrounding ECM [12].

Interferons (IFNs) are a group of cytokines that elicit pleiotropic biological effects, including immunomodulatory, cell differentiative, anti-angiogenic, anti-proliferative, anti-fibrogenic effects [13–15]. IFN-γ is a sole type II IFNs and mediate their effects by binding to cell surface receptors, consisting of IFN-γR1 and IFN-γR2 subunits [16, 17] and thus activating members of the non-receptor tyrosine kinases Janus-activated kinase (JAK)1 and JAK2. Activation of JAKs phosphorylates the signal transducers and activators of transcription (STAT) family of transcription factors. The JAK/STAT signal activated by IFN-γ induce expression the transcription factor IFN response factor 1 (IRF-1) and then regulate the various target genes, including apoptosis-related caspases, Fas/CD95, Fas ligand, and Bcl-2 [18, 19]. The anti-fibrotic effects of interferon-gamma (IFN-γ) are well established in hepatic fibrosis models. It has been reported that IFN-γ has anti-fibrotic effects on hepatic stellate cells, which play important roles in ECM production in the liver by suppressing ECM production regulated by mothers against decapentaplegic homolog 3 (Smad3), Smad7, Y-box-binding protein 1, or p300/CREB-binding protein [20–24]; inhibition of fibroblast–myofibroblast differentiation [25, 26]; and growth retardation of myofibroblasts [27–30]. In addition, IFN-γ modulates tryptophan metabolism by inducting and activating indoleamine 2,3 dioxygenase (IDO), which inhibits ECM production in human dermal fibroblasts [31]. IDO is a heme-containing enzyme that, in humans, is encoded by the IDO1 gene or the variant IDO2 gene [32, 33] and plays roles in inhibiting ECM production, suppressing inflammatory responses, and promoting immune tolerance [31, 34]. However, no consensus has been reached regarding the role of IFN-γ or whether it is harmful or protective with respect to cardiac remodeling [35, 36]. Moreover, no obvious treatment option is available to reduce cardiac fibrosis [36]. Therefore, we investigated whether IFN-γ shows anti-fibrotic effects in human cardiac myofibroblasts (hCMs) in vitro and whether IDO inhibition, which is induced by IFN-γ, would decrease the viability of hCMs.

## Materials and methods

### Cell culture

hCMs, isolated from normal adult ventricles, were purchased from Lonza (Walkersville, MD, USA) and maintained in fibroblast growth medium (FGM)-3 with 10% fetal bovine serum, growth factors (recombinant human fibroblast growth factor and insulin), and gentamicin/amphotericin-B. All cell culture media and supplements were purchased from Lonza. IFN-γ (100 ng/ml; R&D Systems, Minneapolis, MN, USA) or 1-MT (0.5 mM; 1-MT (Sigma, San Diego, CA, USA) were used in the experiments. In this study, P4 hCMs were used.

### Methylthiazolyldiphenyl-tetrazolium bromide (MTT) assays

hCMs (3500 cells/cm^2^) were plated in 96-well plates. After 24 h, cells were treated with IFN-γ or 1-MT for the indicated time points and then MTT (Sigma), dissolved in phosphate-buffered saline (PBS), was added to each well at a final concentration of 5 mg/ml. The hCMs were incubated at 37 °C for 2 h. Formazan (formed in the plates during the assay) was dissolved in 100 μl DMSO, and a microplate reader (BioTek Instruments, Winooski, VT, USA) was used to read the optical density of each well at 570 nm.

### Cell cycle analysis

The Cycle TEST Plus DNA Reagent Kit (BD Biosciences, San Jose, CA, USA) was used to analyze the cellular DNA contents, as per the manufacturer’s instructions. hCMs were trypsinized and then trypsinization was neutralized by adding FGM-3. Next, hCMs were centrifuged at 1800 rpm for 5 min. The cells were washed twice using the wash buffer provided in the kit. Thereafter, the cells were sequentially treated with solutions A, B, and C in a dark room, in compliance with the manufacturer’s instructions. Cellular DNA contents were analyzed by flow cytometry (BD FACSAria III, BD Biosciences).

### Immunoblotting

hCMs were treated with IFN-γ alone or IFN-γ + 1-MT for increasing amounts of time. hCMs were lysed with 1X Laemmli sample buffer (62.5 mM Tris–HCl [pH 6.8], 10% glycerol, 1% sodium dodecyl sulfate [SDS], and 5% β-mercaptoethanol) and boiled for 5 min. Proteins were separated by SDS–polyacrylamide gel electrophoresis (SDS-PAGE). Separated proteins were transferred to an Immobilon-P PVDF membrane (Millipore, Billerica, MA, USA). Then, the membrane was blocked for 30 min in Tris-buffered saline containing 5% non-fat and 0.05% Tween-20, followed by incubation with a primary antibody against IDO1, IDO2, IRF-1, Fas, Fas ligand (FasL), glyceraldehyde 3-phosphate dehydrogenase (Santa Cruz Biotechnology, Santa Cruz, CA, USA), cellular (ED-A) fibronectin (Sigma), or α-smooth muscle actin (SMA) (Abcam, Cambridge, MA, USA). Species-specific horseradish peroxidase-conjugated secondary antibodies (Santa Cruz Biotechnology) were used to detect bound primary antibodies. Visualization of protein bands was performed with the West Pico chemiluminescent substrate (Thermo Scientific, Rockford, IL, USA) and the BioSpectrum imaging system (UVP, Upland, CA, USA).

### Measurement of IDO activity

IDO activities of hCMs were determined by detecting the conversion of tryptophan to kynurenine [37, 38]. Cells were treated with IFN-γ ± 1-MT for 48 or 72 h. Briefly, the cell pellets were washed twice in cold PBS. The cells were re-suspended at 1.5 × 10^7^ cells/ml in PBS and frozen at − 80 °C until being analyzed. Cells were rapidly thawed at 37 °C and refrozen in liquid nitrogen, and this process was repeated three times. Cell lysates (250 μl) were mixed with an equivalent volume of 2 × IDO reaction buffer (100 mM potassium phosphate buffer [pH 6.5], 20 μM methylene blue, 40 mM ascorbate, 200 U/ml catalase, and 800 μM l-tryptophan) and incubated at 37 °C for 30 min. The reaction was ceased by adding 30% trichloroacetic acid (Sigma) and then incubated at 50 °C for 30 min. One hundred microliters of each supernatant were transferred to a microfuge tube after pelleting the proteins at 3000×*g* for 10 min. An equal volume of Ehrlich’s reagent (2% p-dimethylaminobenzaldehyde in glacial acetic acid) was added, and the resulting mixture was incubated at room temperature for 10 min. Kynurenine products were detected at 490 nm using a microplate reader (BioTek Instruments).

### Annexin-V and 7-aminoactinomycin (7-AAD) staining

Apoptosis was measured with the PE-Annexin-V Apoptosis Detection Kit I (BD Biosciences) according to the manufacturer’s instructions. hCMs (3500 cells/cm2) were seeded in 60-mm dishes. After 24 h, cells were treated with IFN-γ or 1-MT for 72 h. hCMs were harvested, washed twice in cold PBS, and re-suspended in 1 × binding buffer. Then, hCMs were stained with PE-annexin-V and 7-AAD at room temperature for 15 min in the dark. Cells were rapidly analyzed without washing by flow cytometer within 1 h. To calculate the dead cell population, the percentages of early (Q4; PE-annexin-V/7-AAD, ±) and late apoptotic cells (Q2; PE-annexin-V/7-AAD, +/+) were analyzed.

### Statistics

Data are expressed as the mean ± standard error (SE). Differences between groups were analyzed by one-way analysis of variance with Tukey’s test against the control. Statistical analysis was performed using SPSS software, version 22.0 (IBM Corporation, Armonk, NY, USA). Statistical significance was defined as *P* < 0.05.

## Results

### Growth retardation of hCMs by IFN-γ

**Fig. 1:**
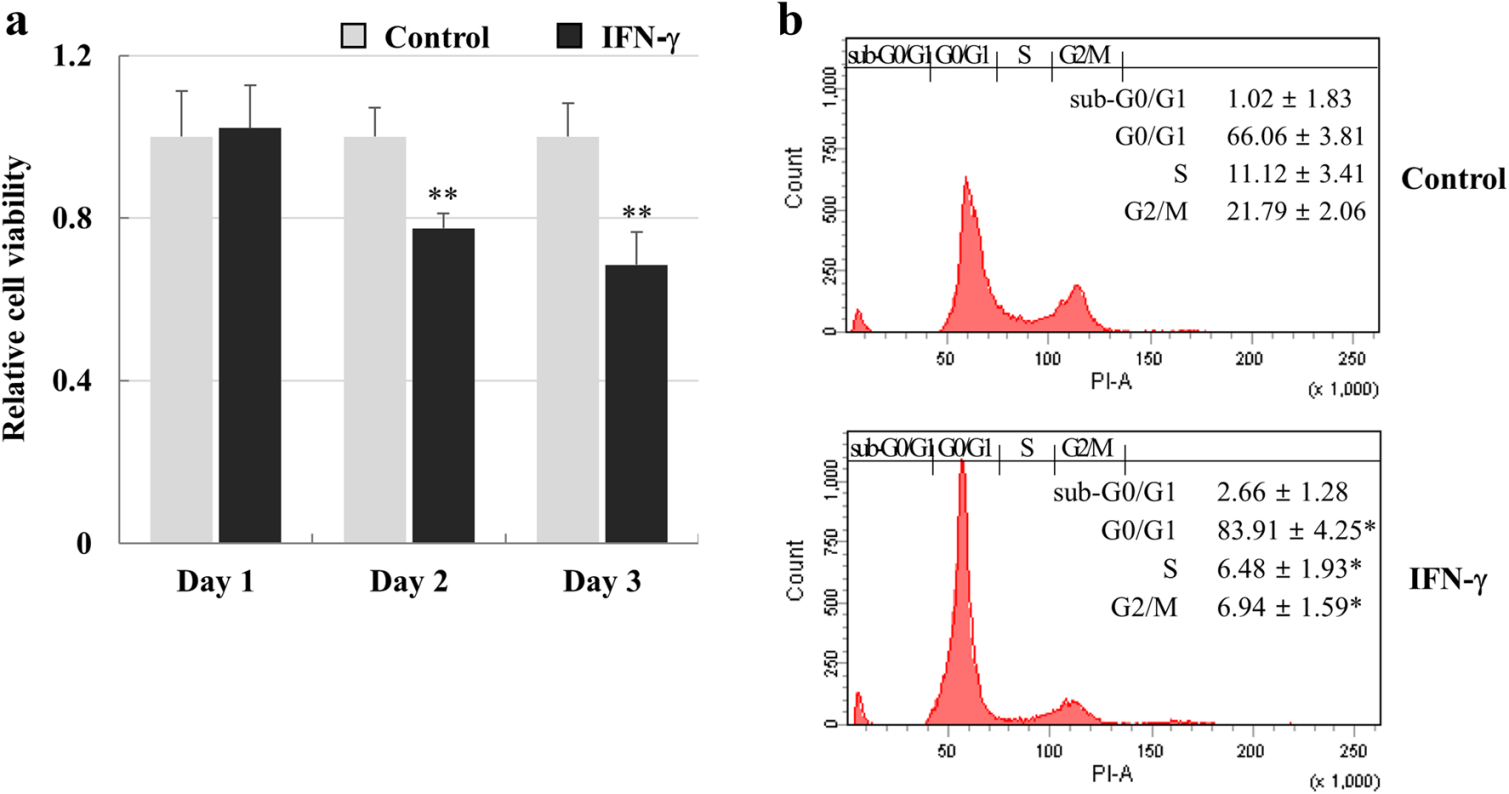
Growth suppression of hCMs by IFN-γ. **a** Cell viability of hCMs treated with IFN-γ. hCMs were treated with 100 ng/ml IFN-γ. Cell viability was evaluated by performing MTT assays. Data are expressed as the mean ± SE of triplicate experiments. **P < 0.01 **b** Cell cycle analysis of hCMs treated with IFN-γ for 72 h. Cellular DNA contents were analyzed by flow cytometry. The data shown are from one of three independent experiments. *P < 0.05

hCMs were treated with 100 ng/ml IFN-γ for 3 days to investigate the growth-inhibitory effect. Cell viability significantly decreased by approximately 22.5% at 2 days and 31.5% at 3 days (Fig. 1a). After treatment with IFN-γ for 3 days, cells in S and G2/M phase decreased in number, but the percentage of the G0/G1 population increased from 66.1 to 83.9% versus the control group. However, the sub-G0/G1 population was not increased by IFN-γ (Fig. 1b). These results suggest that cell cycle arrest in the G0/G1 phase by IFN-γ induced hCM growth retardation.

### IDO induction by IFN-γ in hCMs

**Fig. 2:**
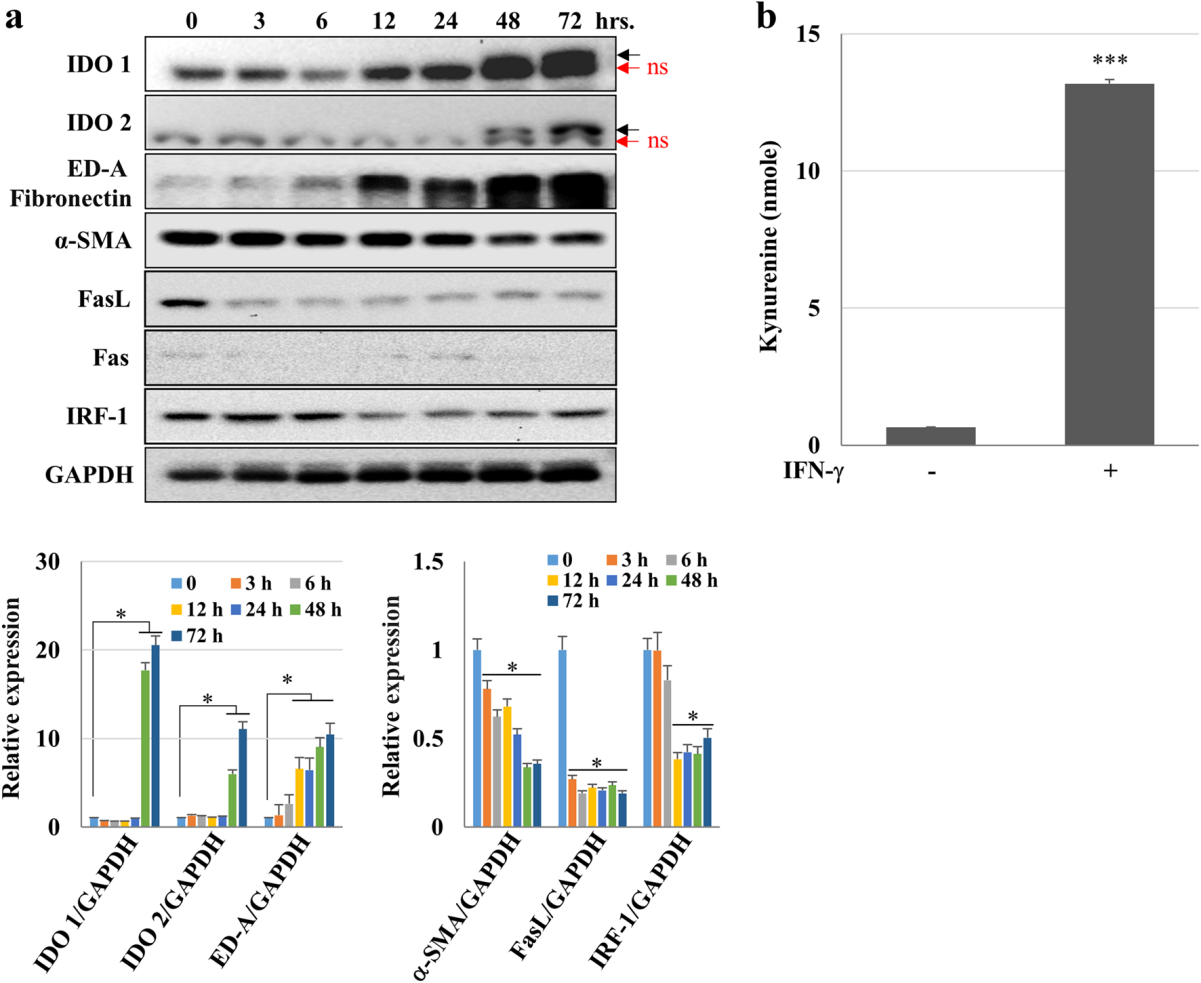
Signaling pathways activated by IFN-γ in hCMs. **a** hCMs were treated with IFN-γ (100 ng/ml) for the indicated time points, and the expression of proteins related to apoptosis (i.e., IRF-1, Fas, and FasL) or cell cycle arrest (i.e., IDO1 and IDO2) was detected by immunoblotting. In addition, STAT1 phosphorylation and α-SMA expression were also evaluated. *P < 0.05 Black arrows: IDO bands; red arrows: non-specific (ns) bands. **b** Kynurenine production in hCMs treated with IFN-γ for 72 h. The data shown are expressed as the mean ± SE of triplicate experiments. ***P < 0.001. (Color figure online)

We investigated whether IFN-γ could induce IDO expression and tryptophan depletion in hCMs. The IDO1 and IDO2 expression levels were elevated at 48- and 72-h post-treatments with IFN-γ. ED-A fibronectin increased in a time-dependent manner, but α-SMA expression gradually decreased. The expression levels of apoptosis-related genes such as IRF-1 and FasL were also diminished, although IRF-1 tended to recover somewhat after 24 h (Fig. 2a). To analyze the IDO enzyme activity after IFN-γ treatment, kynurenine production from hCMs treated with IFN-γ for 72 h was calculated. An average of 13 nmoles of kynurenine was produced by IFN-γ treatment for 72 h (Fig. 2b). These data suggest that IFN-γ induce IDO expression, resulting in tryptophan depletion.

### Cell death after co-treatment with IFN-γ and 1-MT in hCMs via IRF-1, Fas, and FasL expression

**Fig. 3:**
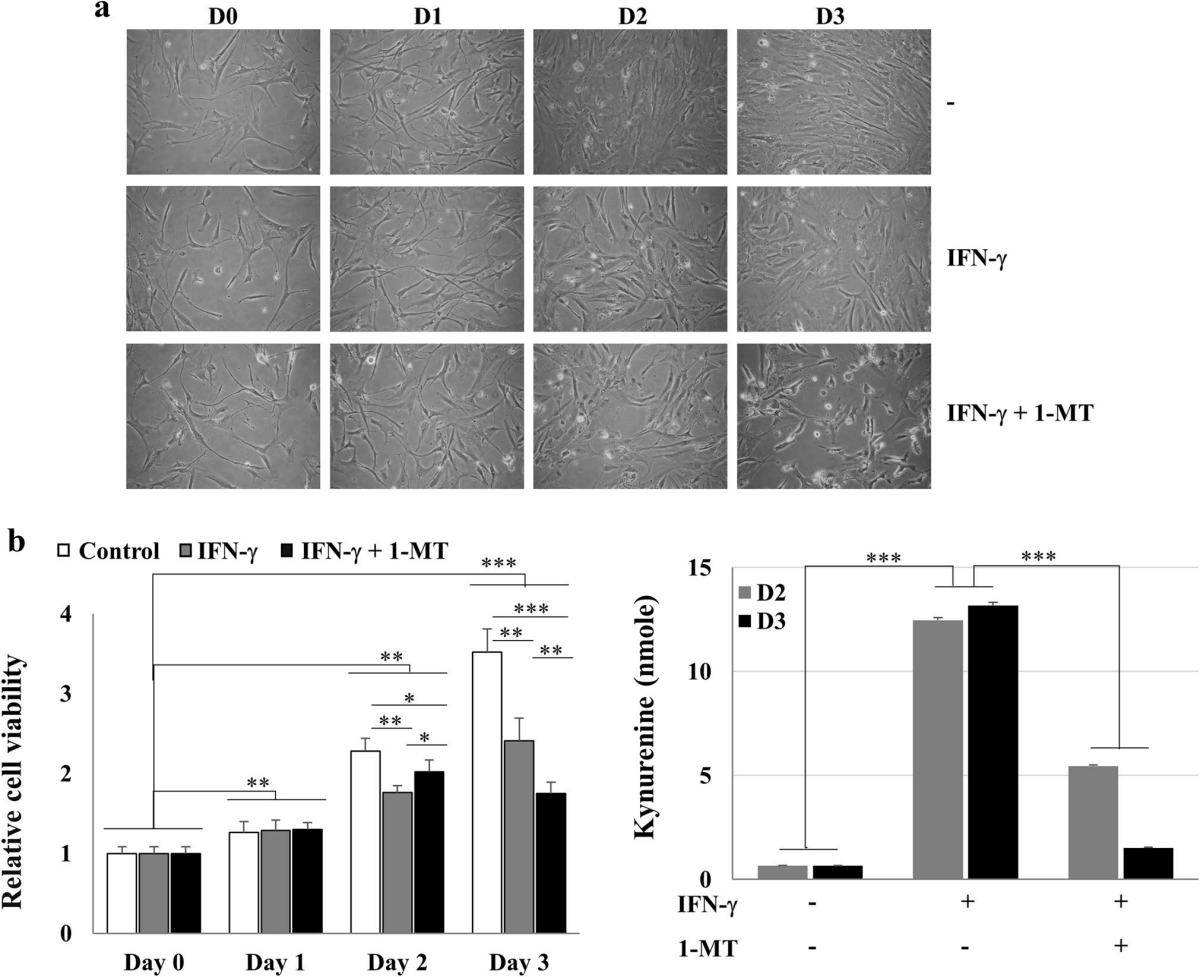
Release from IFN-γ-induced cell cycle arrest by inhibiting IDO activity. **a**, **b** Growth-inhibitory effects of IFN-γ (100 ng/ml) and/or 1-MT (0.5 mM) in hCMs. hCMs were treated with IFN-γ or IFN-γ + 1-MT for the indicated time points, and cell viability was examined by light microscopy (**a**) or MTT assays (**b**). *P < 0.05, **P < 0.01, and ***P < 0.001 **c** Kynurenine production in hCMs treated with IFN-γ or IFN-γ + 1-MT for 48 or 72 h. The data shown are expressed as the mean ± SE of triplicate experiments. ***P < 0.001

Next, we investigated whether inhibiting IDO activity by 1-MT could restore cell growth. Cell death was observed under a microscope after hCMs had been treated with IFN-γ + 1-MT for 3 days (Fig. 3a). Interestingly, growth retardation by IFN-γ was partially reversed by 1-MT on day 2, but cell viability was further reduced by 1-MT treatment on day 3 (Fig. 3b). Moreover, IFN-γ-derived kynurenine production was significantly reduced by 1-MT (Fig. 3c). These results suggest that 1-MT inhibited tryptophan depletion, thereby partially reversing the growth-inhibitory activity of IFN-γ, but eventually induced cell death regardless of tryptophan depletion.

**Fig. 4:**
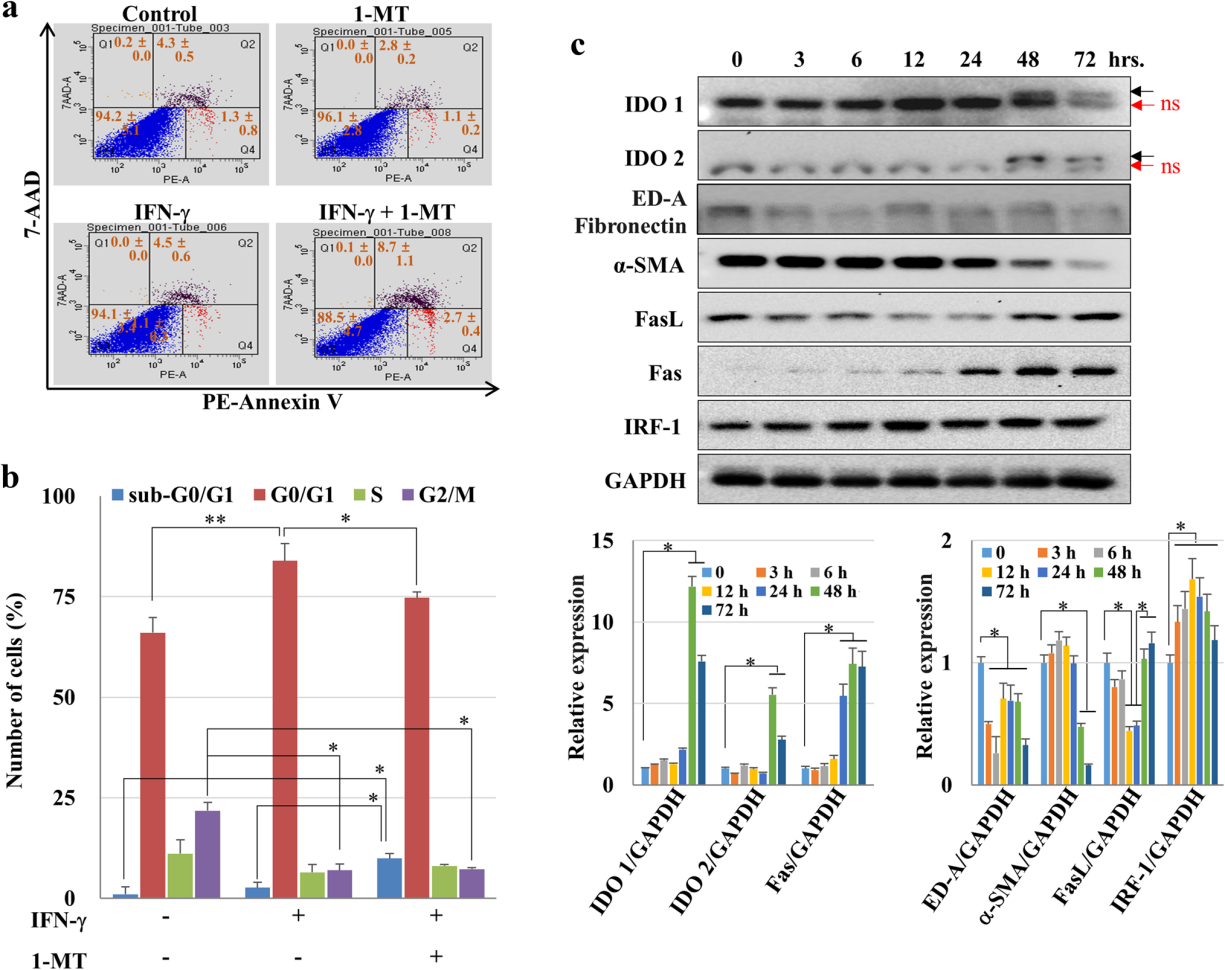
Cell death induced by IFN-γ and 1-MT in hCMs. **a** Cell death of hCMs induced by IFN-γ and 1-MT. hCMs treated with IFN-γ + 1-MT for 72 h were analyzed by PE-annexin-V and 7-AAD staining, and cell death was quantified by flow cytometry. Necrotic (PE-annexin-V/7-AAD, ±), late apoptotic (PE-annexin-V/7-AAD, +/+), live (PE-annexin-V/7-AAD, –/–), and early-apoptotic cells (PE-Annexin-V/7-AAD, ±) are shown in Q1–Q4, respectively. **b** Change in the cell cycle phase induced by IFN-γ + 1-MT. Cellular DNA contents were analyzed by flow cytometry. The data shown are expressed as the mean ± SE of triplicate experiments. *P < 0.05 and **P < 0.01 **c** Signaling pathways activated by IFN-γ and 1-MT in hCMs. In hCMs treated with IFN-γ (100 ng/ml) and 1-MT (0.5 mM) for the indicated time points, the expression of apoptosis-related proteins (i.e., IRF-1, Fas, and FasL) were analyzed by immunoblotting. The y axis of the lower left graph is the logarithmic scale. *P < 0.05 and **P < 0.01. Additionally, p-STAT1, α-SMA, IDO1, and IDO2 were also detected. Black arrows: IDO bands; red arrows: non-specific (ns) bands. (Color figure online)

To confirm that cell death occurred in hCMs treated with IFN-γ and 1-MT, PE-annexin-V/7-AAD staining was performed and DNA contents were analyzed by flow cytometry. The percentage of early (PE-annexin-V-PE/7-AAD, ±) and late apoptotic cells (PE-annexin-V/7-AAD, +/+) increased after co-treatment with IFN-γ and 1-MT to 11.5%, compared with 5.8% in the control group (Fig. 4a). The sub-G0 population also increased from 1 to 10% (Fig. 4b). In addition, α-SMA, ED-A fibronectin, IDO1, and IDO2 expression decreased markedly in hCMs co-treated with IFN-γ and 1-MT compared to that of the IFN-γ-treated group (Fig. 2a). On the other hand, IRF-1 expression increased until 12 h and then gradually decreased to 72 h. But, the IRF-1 expression level was still higher than baseline. Fas, an apoptosis-related-gene, gradually increased over time (Fig. 4c). In addition, although FasL was reduced by 24 h, the expression level was restored at 48 and 72 h. These results suggest that 1-MT modulated the activity of IDO; induced apoptosis in myofibroblasts via the expression of apoptosis-related genes such as IRF-1 and Fas and/or FasL; and consequently reduced fibrosis.

## Discussion

Activated CMs lead to myocardial scarring and fibrosis of damaged or diseased myocardium by regulating cardiac ECM production [39]. Therefore, CM regulation is important for ameliorating cardiac remodeling. We observed that (1) IFN-γ inhibited hCM proliferation through G0/G1 cell cycle arrest and reduced the expression of α-SMA, a marker of fibrosis [40]. (2) G0/G1 cell cycle arrest was induced by tryptophan depletion through increased IDO activity, induced by IFN-γ. (3) Co-treatment with IFN-γ and IDO inhibitor (1-MT) markedly reduced the activity of hCMs expressing α-SMA and induced apoptosis through up-regulating the IRF-1, Fas, and FasL genes.

Conflicting data have been reported regarding whether IFN-γ is harmful or protective for the heart. IFN-γ overexpression caused chronic active myocarditis, eventually resulting in cardiomyopathy in IFN-γ transgenic mice [41]. Marko et al. [42] reported that IFN-γ blockade reduced inflammation and cardiac fibrosis in IFN-γ receptor-knockout mice. Han et al. [43] also demonstrated that IFN-γ knockout reduced the accumulation of α-SMA-positive cells and α-SMA expression in an angiotensin II-induced cardiac fibrosis model. In contrast, Fairweather et al. [44] reported that IFN-γ deficiency led to increased chronic viral myocarditis following cardiac fibrosis, pericarditis, and dilated cardiomyopathy in IFN-γ-deficient mice. Mast cell degranulation and profibrotic cytokines (i.e., transforming growth factor-β_1_, interleukin-1β, and interleukin-4) were increased in the heart. Moreover, Afanasyeva et al. found that IFN-γ deficiency increased cardiac inflammation and resulted in dilated cardiomyopathy and heart failure in a mouse model of autoimmune myocarditis [45].

Despite these conflicting results, little information has been reported regarding whether IFN-γ can directly regulate the proliferation and activity of CMs. IFN-γ is well known to express IDO, a rate-limiting enzyme of tryptophan catabolism. Consequently, degradation of the essential amino acid tryptophan to kynurenine causes cell starvation and induces anti-proliferative effects for pathogens, tumor cells, immune cells, and mesenchymal stem cells [46–49], as well as the hCMs used in this study. In addition, tryptophan-derived catabolites including kynurenine inhibited activated T cell and natural killer cell proliferation [50].

Interestingly, co-treatment with IFN-γ and 1-MT induced cell death in hCMs rather than the recovery of proliferative activity. The underlying mechanism is not fully understood. IFNs regulate the expression of interferon regulatory factor protein family members, including IRF-1. IRF-1 regulates the expression of target genes including *IDO* by binding to an interferon-stimulated response element in their promoters. IRF-1 can also modulate the expression of FasL and induce apoptosis in T cells [51]. In our systems, IFN-γ reduced IRF-1 expression, but co-treatment with IFN-γ and 1-MT gradually increased the expression of IRF-1 by 12 h. However, after 12 h, these expression levels decreased but remained at levels comparable to those of the control group at 72 h. In addition, Fas expression markedly increased in hCMs co-treated with IFN-γ and 1-MT, but FasL expression decreased by 24 h before returning to an increased level by 48 and 72 h. These results suggest that the role of IRF-1 in the expression of IDO and Fas may be different in hCMs. In other words, IFN-γ can induce IRF-1-independent IDO expression, thereby promoting G0/G1 cell cycle arrest through tryptophan depletion in hCMs. However, the IDO inhibitor 1-MT may increase IRF-1-dependent Fas expression and induce apoptosis of hCMs..

Because this in vitro study was performed at the cellular level, in vivo animal models are needed to confirm these results. However, to our knowledge, this is the first study to demonstrate the regulation of activated CMs by IFN-γ and 1-MT. In the future, it should be confirmed whether co-treatment with IFN-γ and 1-MT can control cardiac fibrosis in animal and human models.

In summary, we report that IFN-γ-induced IDO expression decreased cell growth and induced G0/G1 cell cycle arrest in hCMs through tryptophan depletion. Moreover, inhibition of IDO expression with the IDO inhibitor 1-MT increased apoptosis in hCMs through the induction of Fas, FasL, and IRF-1.
